# TGFA expression is associated with poor prognosis and promotes the development of cervical cancer

**DOI:** 10.1111/jcmm.18086

**Published:** 2023-12-28

**Authors:** Xiaoxuan Ma, Jingying Zheng, Kang He, Liangjia Wang, Zeyu Wang, Kai Wang, Zunlong Liu, Zhiqiang San, Lijing Zhao, Lisheng Wang

**Affiliations:** ^1^ Department of Rehabilitation School of Nursing Jilin University Changchun China; ^2^ Department of Gynecology and Obstetrics Second Hospital of Jilin University Changchun China

**Keywords:** bioinformatics, biomarker, cervical cancer, prognosis, TGFA

## Abstract

Cervical squamous cell carcinoma and endocervical adenocarcinoma (CESC) are the second most common cancers in women aged 20–39. While HPV screening can help with early detection of cervical cancer, many patients are already in the medium to late stages when they are identified. As a result, searching for novel biomarkers to predict CESC prognosis and propose molecular treatment targets is critical. TGFA is a polypeptide growth factor with a high affinity for the epidermal growth factor receptor. Several studies have shown that TGFA can improve cancer growth and progression, but data on its impact on the occurrence and advancement of CESC is limited. In this study, we used clinical data analysis and bioinformatics techniques to explore the relationship between TGFA and CESC. The results showed that TGFA was highly expressed in cervical cancer tissues and cells. TGFA knockdown can inhibit the proliferation, migration and invasion of cervical cancer cells. In addition, after TGFA knockout, the expression of IL family and MMP family proteins in CESC cell lines was significantly reduced. In conclusion, TGFA plays an important role in the occurrence and development of cervical cancer. Therefore, TGFA may become a new target for cervical cancer treatment.

## 1 INTRODUCTION

Cervical squamous cell carcinoma and endocervical adenocarcinoma (CESC) are the second‐largest causes of cancer death in women aged 20–39 years, according to Cancer Statistics 2022.^1^ High‐risk human papillomavirus (HPV) infection remains a major risk factor for cervical cancer.^2^ At the moment, the HPV vaccine offers considerable protection against cervical cancer.^3^ However, a significant percentage of women have passed the vaccination age and frequently lack access to the HPV vaccine in places with lower socioeconomic levels. This will lead to hundreds of thousands of deaths from cervical cancer in the future.^4^ As a result, discovering novel biomarkers to predict patient prognosis and suggest treatment targets is critical.

Transforming growth factor α (TGFA) belongs to the epidermal growth factor (EGF) family of polypeptide growth factors and has a high affinity with the epidermal growth factor receptor (EGFR).^5^ When combined, EGF receptor (EGFR) dimer and tyrosine kinase activities are activated, and then downstream signalling pathways such as RAS/Raf/MAPK and PI3K/AKT are activated to promote cell proliferation, invasion, inhibit apoptosis and promote angiogenesis.^6^, ^7^, ^8^, ^9^, ^10^, ^11^ TGFA may be associated with a variety of diseases. Mutation of the TGFA gene may increase the incidence of non‐syndromic stomatofacial fissure and knee osteoarthritis.^12^, ^13^, ^14^ TGFA signalling encourages the development of lung fibrosis in mice models.^15^, ^16^ TGFA has been shown to contribute to the malignant behaviour of breast cancer cells in vitro and in vivo.^17^ Studies have found that TGFA is considered to promote colon cancer migration and invasion by regulating epithelial‐mesenchymal transition (EMT) markers and the NF‐κB signalling pathway.^18^ In many other tumours, including gastric cancer and melanoma, TGFA expression is up‐regulated, and TGF‐EGFR signalling may promote tumour development through the autocrine growth factor pathway.^19^ These studies suggest that TGFA may promote cancer growth and progression. However, data on the role of TGFA in the occurrence and progression of CESC are limited.

In this work, TGFA expression and its associations with clinical characteristics were determined using the Cancer Genome Atlas (TCGA) and Gene Expression Atlas (GEO) datasets. Immunohistochemical staining revealed TGFA expression in 16 normal cervix, 6 cases of cervical precancerous lesions (CIN‐III) and 42 cases of cervical cancer samples. To anticipate the function of TGFA in the formation of CESC and its influence on patient prognosis, researchers employed Gene Ontology (GO), Kyoto Encyclopedia of Genes and Genomes (KEGG), gene set enrichment analysis (GSEA) and immunoinfiltration analysis. Meanwhile, protein–protein interaction (PPI) networks based on TGFA and its associated differentially expressed genes were built. Finally, HeLa and SiHa cells were used to investigate the role of TGFA in cell proliferation, metastasis and invasion.

## 2 METHODS AND MATERIALS

### 2.1 Preprocessing and Data Sources

The GTEx database and RNA‐seq data from the TCGA CESC project (https://portal.gdc.cancer.gov/) explain TGFA differential expression in unpaired and paired samples and the Toil process uniformized the data.^20^ The log2‐transformed TCGA level 3 HTSeq‐FPKM (Fragments Per Kilobase Per Million) format was translated to the TPM (transcripts per million reads) format. TPM‐formatted data were used in all final TCGA‐based studies. Using GEO query [version 2.54.1],^21^ the differential analysis data for TGFA in datasets GSE6791 and GSE7410 were extracted from the GEO database.^22^, ^23^ These results were produced by deleting probes that were associated with several compounds, and when multiple probes from the same molecule were detected, only the probe with the highest signal value was retained. The data were then normalized once more using the normalize Between Arrays function of the limma package [version 3.42.2].^24^ In R (version 3.6.3), all statistical computations and visualisations were carried out.

### 2.2 Single‐gene differential analysis and correlation analysis of TGFA

The DESeq2 package [version 1.26.0] and the STAT package [version 3.6.3] were used to conduct single‐gene differential analysis and single‐gene correlation analysis of TGFA in the CESC project utilising the TCGA database.^25^ Using the ggplot2 program [version 3.3.3], the results of the single‐gene differential analysis were shown as volcanoes. The |log_2_ fold change (LogFC)| > 1 and *p*_adj_ <0.05 criteria were used to identify differentially expressed genes (DEGs). The STRING database was utilized to show the DEGs,^26^ the Cytoscape tool was used to investigate the PPI network of DEGs, and the MCODE plugin was used to determine the HUB genes. The top 35 correlations were found when the genes from the single‐gene correlation research were ordered by |Pearson value| in descending order. The ggplot2 [version 3.3.3] program was used to produce the TGFA single‐gene co‐expression heatmap utilising the leading 35 genes and the HUB gene.

### 2.3 Analysis of Functional Enrichment

In order to evaluate putative signalling pathways in the light of differential expression analysis (TGFA up‐regulation vs. TGFA down‐regulation samples), the TCGA CESC study used gene set enrichment analysis (GSEA). The threshold for significant enrichment was an adjusted *p* value of 0.05. The R packages ‘clusterProfiler’ and ‘org.Hs.eg.db’ were used to conduct the Gene Ontology (GO) and Kyoto Encyclopedia of Genes and Genomes (KEGG) analyses after screening the DEGs based on the thresholds (|LogFC | > 1 and *p*_adj_ <0.05).If *p*_adj_ <0.05, it was regarded to be significantly enriched.

### 2.4 Immunoinfiltration Analysis of TGFA

GSVA [version 1.34.0] was used to examine the relative infiltration levels of 24 immune cells.^27^ ssGSEA and Spearman correlation analysis were used in the immune infiltration method. Bindea et al. proposed 24 immune cell markers.^28^ Following that, the samples were divided into two groups: TGFA down‐regulated and TGFA up‐regulated. The GSVA software [version 1.34.0] was used to calculate the enrichment percentage of different immune cell infiltrations in each subgroup.

### 2.5 Analysing the connection between the prognosis of CESC patients and the expression of TGFA mRNA

CESC patients' survival data were statistically analysed with the survival package [version 3.2‐10], and the results were plotted on Kaplan–Meier curves with the survminer package [version 0.4.9] to plot overall survival (OS), disease‐specific survival (DSS) and progression‐free interval (PFI).^29^ ROC analysis was done on the data using the pROC tool [version 1.17.0.1] to analyse the accuracy of TGFA for prognostication. Prognostic data from the survival study presented by Liu et al. were used for all previous statements.^30^ Finally, a binary logistic regression model was established to predict the relationship between different clinicopathological features and TGFA expression.

### 2.6 Specimens

The Jilin University School of Nursing Ethics Review Committee (Changchun, China) gave its approval for this study. The selection procedure comprised choosing human cervical tissue samples that were taken between June and December 2022 from the Obstetrics and Gynecology Department of the Second Hospital of Jilin University (Changchun, China). The inclusion criteria are as follows: (i) pathological diagnosis of cervical cancer, including cervical squamous cell carcinoma and endocervical adenocarcinoma; (ii) complete clinical data, including age, clinical stage, grade, pathological type, invasion and lymph node metastasis^31^; (iii) no history of physical therapy or preoperative chemoradiotherapy. The exclusion criteria are as follows: (i) incomplete case data; (ii) combined immune diseases and other malignancies; (iii) history of adjuvant chemotherapy and radiotherapy before surgery; (iv) use of pathological case data without the informed consent of the patient. Sixty four specimens from the tumour collection were assessed using the fourth version of the ‘IARC WHO Classification of Female Genital Organ Tumors’ issued by the World Health Organization (WHO). Histopathological diagnoses were made by two pathologists, including 16 normal tissue cases, 6 cases of precancerous lesions (CIN III), 40 cervical squamous cell carcinoma cases and 2 cervical adenocarcinoma cases. Patients with a history of other cancers or preoperative anticancer treatment were excluded. All specimens were immunohistochemically stained with unstained paraffin sections and then re‐diagnosed by the same pathologist.

### 2.7 Establishing stable transfection cell lines using cell culture

iCell Bioscience Inc., Shanghai, provided the human CESC cell lines HeLa and SiHa. DMEM (including 10% FBS, 1% penicillin and streptomycin) was used to cultivate HeLa cells. SiHa cells were grown in MEM (10% FBS, 1% penicillin and 1% streptomycin). The two cell lines were grown in a 37°C environment with 5% CO_2_, with the medium replaced every 2 days.

The lentiviral vector plasmid pLKO.1‐Puro (product code FH1717; Hunan Fenghui Biotechnology Co., Ltd.) was utilized to construct the pLKO.1‐Scramble and pLKO.1‐shTGFA plasmids. The interference sequence of shTGFA is 5′‐CCAACACAGGAGATTTCTATA‐3′, and the interference sequence of shScramble is 5′‐GCTTCGCCGTAGTCTTA‐3′. In this study, a 3‐plasmid packaging system was used for lentivirus packaging.^29^ After combining the lentiviral vector plasmids with the packaging plasmid PMD2.G (product code BR037, Fenghui), psPAX2 (product code BR036, Fenghui) and Lipofectamine™ 3000 transfection reagent (product code L3000150, Thermo Fisher), the complexed solution was introduced to HEK‐293 T cells (product code iCell‐h237, iCell). After 48 and 72 h, the medium was collected and filtered with a 0.22 μm filter. The virus titre was detected using a Lentivirus titre rapid assay card (colloidal gold method) (G1804‐10 T, Servicebio) according to the manufacturer's instructions (virus titre greater than 1.25 × 10^6^ TU/mL). The medium was kept at 4°C for a week and then at −80°C.

Cells (300,000 cells/well) were seeded in six‐well plates and cultured in medium for 24 h before lentivirus infection. When the cells were 30%–40% confluent, lentivirus of shTGFA or shScramble was added to each well of HeLa (MOI = 15) and SiHa (MOI = 20) cells using 5 μg/mL of polybrene. After 48 h, the medium was modified. Selected cells were grown in puromycin at a concentration of 2 μg/mL after infection with viruses carrying the puromycin resistance gene. Puromycin selection was continued for a week prior to cell collection and analysis.

### 2.8 Immunohistochemical Staining

According to previous studies,^32^ immunohistochemical (IHC) staining was conducted. After the samples were fixed for 24 h at room temperature in 10% formalin, they were sectioned to a thickness of 3 μm and fixed in paraffin. After soaking in EDTA retrieval buffer, the sections were microwave‐cooked. After that, 5% bovine serum albumin was given to avoid nonspecific binding for 20 min at room temperature. Rabbit anti‐TGFA antibody (AF0262; 1:100; Affinity) was used to stain histological sections overnight at 4°C. The secondary antibody was goat anti‐rabbit IgG conjugated with horseradish peroxidase (GB23204; 1:200; Servicebio), and the staining was done at 37°C for 30 min. Using 3,3′‐diaminobenzidene (Boster Biological Technology, Inc.) as the chromogen, reactive products were seen; moreover, the sections were counterstained with 0.1% haematoxylin (Boster Biological Technology, Inc.) for 2 min at room temperature. The stained slices were imaged using a light microscope with an objective magnification of ×200 or ×400. The positive cell density was evaluated using Image‐Pro Plus 6.0 (Media Cybernetics, Inc.), and the findings are shown as average optical density (AOD) values. In a double‐blind situation, the immunohistochemical staining was evaluated separately by two skilled pathologists from the Bethune Second Hospital of Jilin University's Department of Pathology.

### 2.9 Real‐time PCR

The EasyPure RNA kit (product code ER101‐01; TransGen) was used to extract total RNA from stably transfected cells, and first‐strand cDNA was generated using a cDNA synthesis kit (product code AT311‐02; TransGen) according to the manufacturer's instructions. Real‐time PCR was used to evaluate TGFA transcription levels using the SYBR Green qPCR kit (product code AQ132; TransGen) with GAPDH as the internal reference gene. The following primers were used: TGFA gene, 5′‐GATTCCCACACTCAGTTCTGC‐3′ for forward and 5′‐GAAGATGGTGATGGGATTTC‐3′ for reverse; GAPDH gene, 5′‐GAAGGTGAAGGTCGGAGTC‐3′ for forward and 5′‐GAAGATGGTGATGGGATTTC‐3′ for reverse. An ABI‐Q3 was used to conduct PCR at 94°C for 30 s, followed by 45 cycles of amplification at 94°C for 5 s, 51°C for 15 s and 72°C for 10 s (Thermo Fisher Scientific, Inc.). The mRNA expression level was quantified by 2^−ΔΔCt^.^29^

### 2.10 Western Blotting Analysis

The collected cell samples were cracked on ice using RIPA cracking buffer containing PMSF (Servicebio, China). After centrifugation, the precipitate was removed, and the total cell protein was obtained. The protein concentration of the sample was measured by the BCA protein assay kit (Servicebio, China), and the same protein system was prepared in a certain proportion with the sample buffer (Servicebio, China). The target protein was isolated by SDS‐PAGE gel electrophoresis at 80 V for 10 min and at 150 V for 60 min. The protein was then transferred to the PVDF membrane through a transfer tank at 300 mA for 45 min. Rabbit anti‐GAPDH antibody (1:3000, GB15004, Servicebio), rabbit anti‐TGFA antibody (1:1000, GB112570, Servicebio), rabbit anti‐IL‐1A antibody (1:1000, GB112359, Servicebio), rabbit anti‐IL‐17 antibody (1:1000, GB11110‐1, Servicebio), rabbit anti‐MMP1 antibody (1:1000, 10371‐2‐AP, Servicebio), rabbit anti‐MMP10 antibody (1:1000, GB112994, Servicebio), rabbit anti‐MMP13 antibody (1:1000, GB11247‐1, Servicebio) was incubated overnight at 4°C. The PVDF membrane was washed with TBST for three times for 5 min and then incubated with HRP‐Goat anti‐Rabbit (1:5000, GB23303, Servicebio) for 1 h. The residual secondary antibodies are then washed with TBST. Finally, the target protein bands were visualized using the enhanced chemiluminescence kit (Servicebio, China) and standardized as internal standards.

### 2.11 Assay for Cell Proliferation

Equal numbers of transfected HeLa and SiHa human cells were plated in a 96‐well plate. After 22, 46, and 94 h, CCK‐8 reagent was applied to each well (10 μL/well; Bioss product number BA00208). A microplate reader (E0226; Detie, Inc.) was used to measure the absorbance of each well at 450 nm following 2 h of incubation at 37°C.

Cell proliferation was examined using colony‐formation techniques. Before the appearance of typical colonies, transfected human HeLa and SiHa were cultured in DMEM/MEM under 5% CO_2_ at 37°C in 10% FBS for 10 days. Colony formation methods were used to study cell growth. Transfected human HeLa and SiHa were grown in DMEM/MEM at 37°C in 10% FBS for 10 days prior to the development of typical colonies.

### 2.12 Assay for Wound Healing

To test the migratory ability of the CESC cells mentioned above, a wound‐healing experiment was undertaken. Using a 200‐μL pipette tip to scratch cells cultivated in six‐well plates (3 × 10^5^ cells/well), a linear wound appeared. The dislodged cells were cleaned and eliminated in PBS. To monitor the infiltration of cells into the injured area, pictures were obtained at 24 and 48 h using a digital camera and an optical microscope (Motic Corporation). For each type of cell, micrographs of the same size and duration were taken.

### 2.13 Invasion Assay

A transwell chamber (Labselect, 14342) was utilized for the invasion experiment to test the invasive capability of the CESC cells indicated above. As directed by the manufacturer, Matrigel (BD Biosciences, 356234) was used to coat the chamber. The upper chamber contained 3 × 10^4^ cells in 100 μL of serum‐free DMEM or MEM, while the lower chamber contained 600 mL of 10% FBS media‐based material. The remaining cells on the top surface were removed using a cotton swab following 30 h of treatment at 37°C, and the invasive cells were stained with 10% Giemsa. The photographs were taken with an optical microscope (AE2000, Motic).

### 2.14 Statistical analysis

In the statistical analysis, the standard deviation (SD) plus the mean of three independent tests was used. For statistical studies, SPSS 23.0 and R version 3.6.3 were used; a one‐factor ANOVA was used to examine group differences, followed by Dunnett's post hoc test, Kruskal–Wallis test or Student's *t*‐test. When *p* < 0.05 was used, differences were deemed statistically significant.

## 3 RESULTS

### 3.1 The expression of TGFA in different tissues was analysed by bioinformatics and immunohistochemistry

**FIGURE 1:**
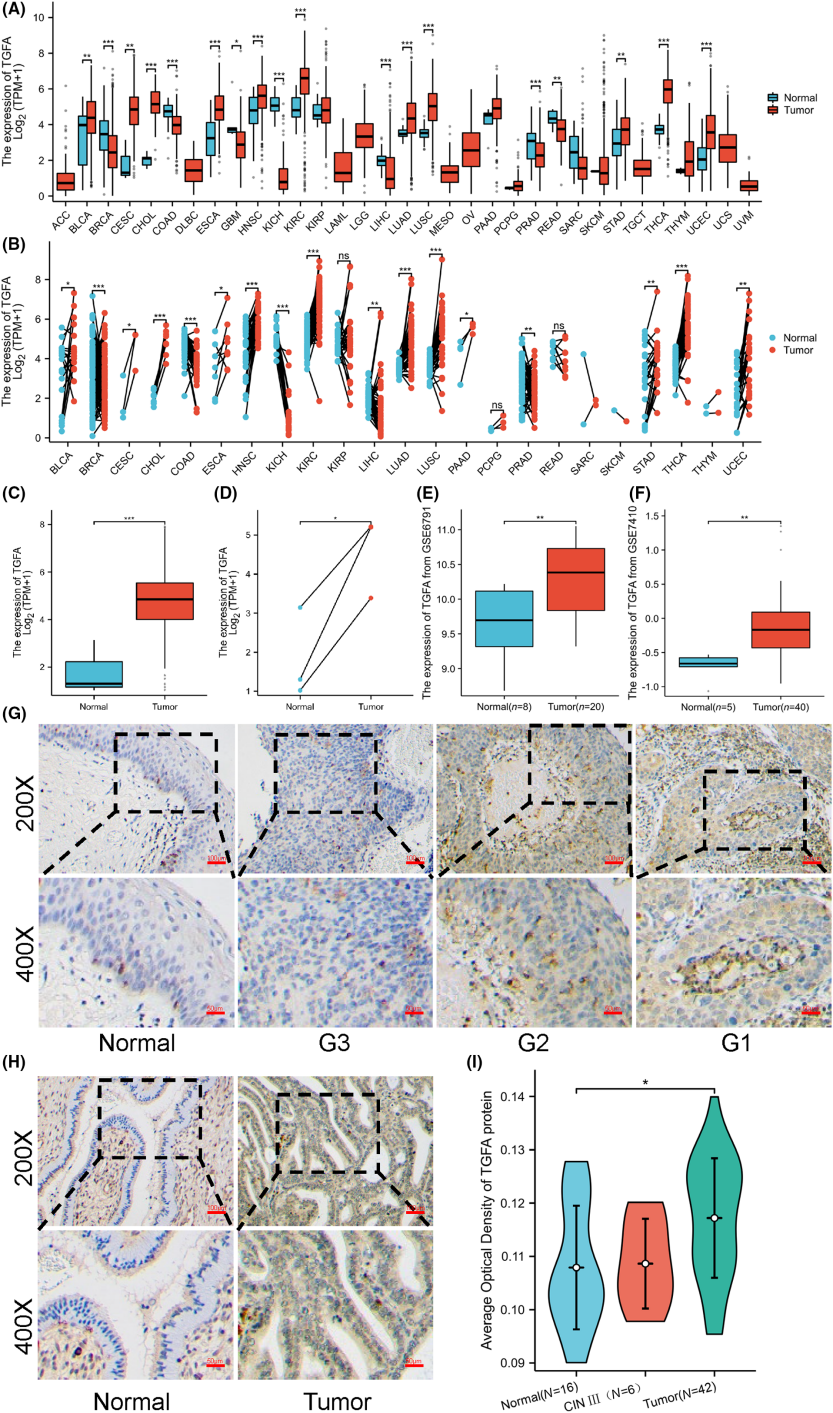
The expression of transforming growth factor α (TGFA) in different patients was analysed by bioinformatics and immunohistochemistry. (A) TGFA expression difference analysis results of 33 tumours based on TCGA database data. (B) Pan‐cancer analysis of paired samples based on TCGA database data. (C) Differential analysis of TGFA expression in unpaired cervical squamous cell carcinoma and endocervical adenocarcinoma (CESC) samples. (D) Differential analysis of TGFA expression in paired CESC samples. (E) TGFA expression differential analysis based on GSE6791 data. (F) TGFA expression differential analysis based on GSE7410 data. (G) Immunohistochemical results of TGFA in normal cervical tissues and cervical squamous cell carcinoma with different degrees of differentiation. (H) Immunohistochemical results of TGFA in normal and adenocarcinoma tissues of the cervix. (I) Group comparison of TGFA immunohistochemical results in 16 normal cervical tissues, 6 precancerous lesions (CIN‐III), and 42 CESC tissues. Significance identifier: ns (no significance), *p* ≥ 0.05; *, *p* < 0.05; **p < 0.01; ***p < 0.001.

Using the TCGA database for bioinformatics analysis, we found that in the unmatched samples, the expression of TGFA was higher in the following malignancies than in normal tissues: bladder urothelial carcinoma (BLCA), cervical squamous cell carcinoma and endometrial adenocarcinoma (CESC), cholangiocarcinoma (CHOL), oesophageal carcinoma (ESCA), head and neck squamous cell carcinoma (HNSC), kidney clear cell carcinoma (KIRC), lung adenocarcinoma (LUAD), and lung squamous cell carcinoma cancer (LUSC), gastric adenocarcinoma (STAD), thyroid adenocarcinoma (THCA) and endometrial cancer (UCEC). Similarly, TGFA expression is also reduced in breast invasive carcinoma (BRCA), colon cancer (COAD), glioblastoma multiforme (GBM), chromophobe tumour of the kidney (KICH), hepatocellular carcinoma (LIHC), prostate adenocarcinoma (PRAD) and rectal adenocarcinoma (READ) compared to normal tissue, as shown in Figure 1A.

In the paired samples, the expression of TGFA in BLCA, CESC, CHOL, ESCA, HNSC, KIRC, LIHC, LUAD, LUSC, PAAD, STAD, THCA and UCEC was higher than that in neighbouring tissues. The expression of TGFA in BRCA, COAD, KICH and PRAD is lower than that in neighbouring tissues, as shown in Figure 1B. The number of tumour samples used in the pan‐cancer analysis is shown in Table S1. In unpaired and paired CESC samples, TGFA expression in tumours was higher than in normal tissues, as shown in Figure 1C,D. Meanwhile, we verified this point by using TGFA mRNA and protein expression data in the GSE6791 and GSE145976 databases, and the results were consistent with those in the TCGA database, as shown in Figure 1E,F.

The expression of TGFA in 16 normal cervical tissues, 6 precancerous lesions (CIN‐III) and 42 CESC tissues was determined by immunohistochemical staining. As shown in Figure 1G, the expression of TGFA protein is low in normal cervical tissues and upregulated in squamous cell carcinoma tissues, and the expression of TGFA protein increases gradually with the decrease of tumour differentiation. Similarly, the expression of TGFA in adenocarcinoma was also significantly increased, as shown in Figure 1H. TGFA expression was not significantly elevated in precancerous tissues of cervical cancer, as shown in Figure S1. The average absorbance statistics of TGFA protein expression levels in 16 normal tissues, 6 precancerous tissues and 42 CESC tissues are shown in Figure 1I, and the expression of TGFA in tumour tissues is significantly increased.

### 3.2 Influence of TGFA expression in CESC on the diagnosis and prognosis of patients

**FIGURE 2:**
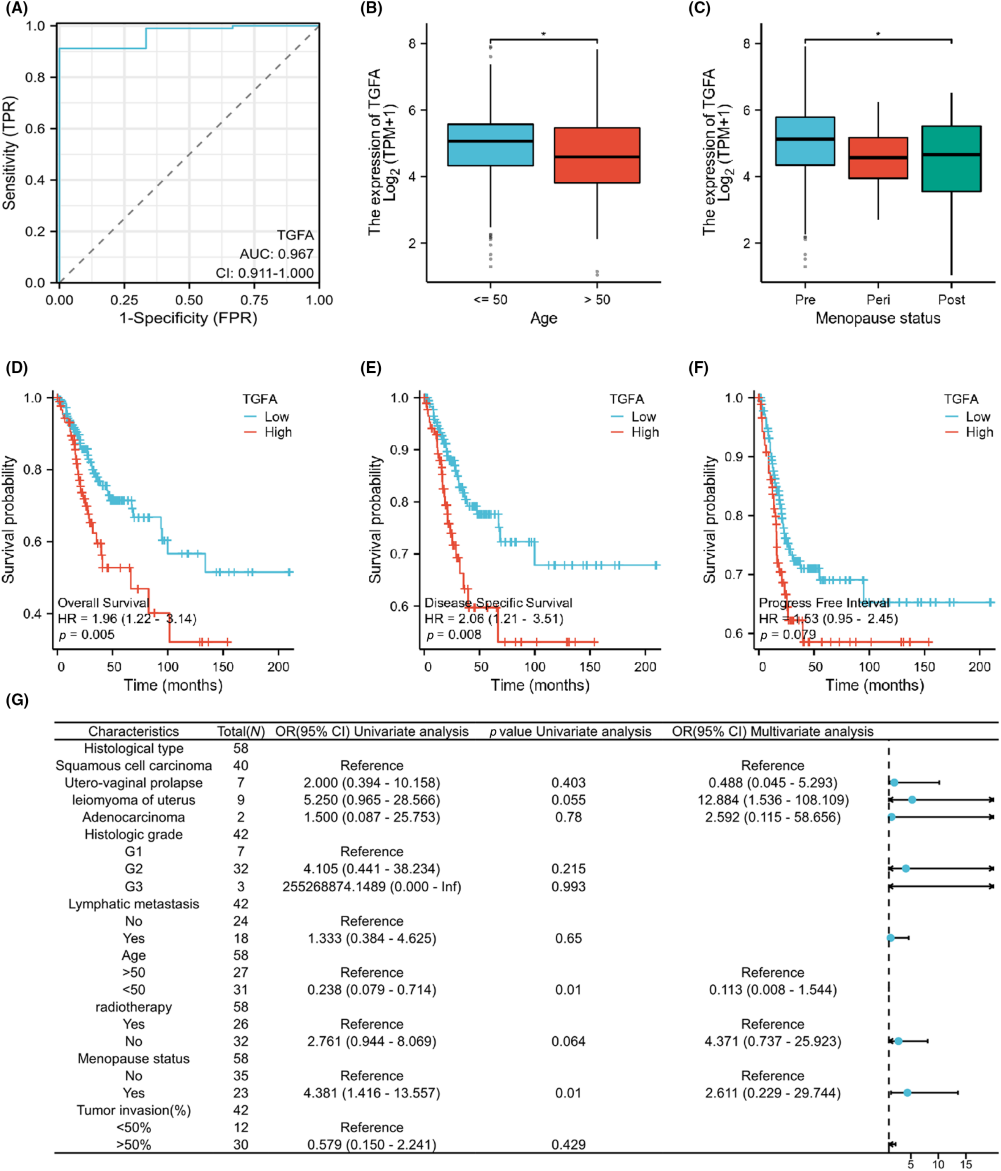
Influence of transforming growth factor α (TGFA) expression in cervical squamous cell carcinoma and endocervical adenocarcinoma (CESC) on diagnosis and prognosis of patients. (A) TGFA diagnostic ROC curve; the area under the ROC curve is between 0.5 and 1. The closer the AUC is to 1, the better the diagnostic effect. When the AUC is between 0.5 and 0.7, the accuracy is low, when the AUC is between 0.7 and 0.9, the accuracy is moderate, and when the AUC is above 0.9, the accuracy is high. (B, C) Clinical significance of TGFA expression with age and menopausal status. (D–F) Stratified KM survival curves for overall survival (OS), disease‐specific survival (DSS), and progression‐free period (PFI) based on TGFA expression. (G) Binary logistic regression analysis results of correlation between TGFA expression level and clinical features in 58 patients. The data is incomplete because some records are missing. Significance identifier: ns (no significance), *p* ≥ 0.05; *p < 0.05; **p < 0.01; ***p < 0.001.

The ROC curve was constructed according to TGFA expression in CESC, and the calculated area (AUC) value under the curve was 0.967, which had a high diagnostic value, as shown in Figure 2A. Furthermore, TGFA expression was significantly correlated with age and climacteric status (*p* < 0.05), as shown in Figure 2B,C, and the clinical baseline data table is shown in Table S2. However, TGFA levels were not associated with a patient's tumour T, N, M, clinical stage or histological tumour type and grade (*p* > 0.05), as shown in Figure S2. In order to further determine the relationship between TGFA expression and the prognosis of CESC patients, we used the prognostic data of CESC in TCGA for survival analysis, and the results are shown in Figure 2D–F. We found that high expression of TGFA was associated with poorer OS, DSS and PFI. After that, we analysed the relationship between TGFA expression and various clinical characteristics of CESC patients, and the results showed that the expression of TGFA was closely related to the age and menopausal status of CESC patients, as shown in Figure 2G.

### 3.3 Analysis of TGFA‐related differentially expressed genes

**FIGURE 3:**
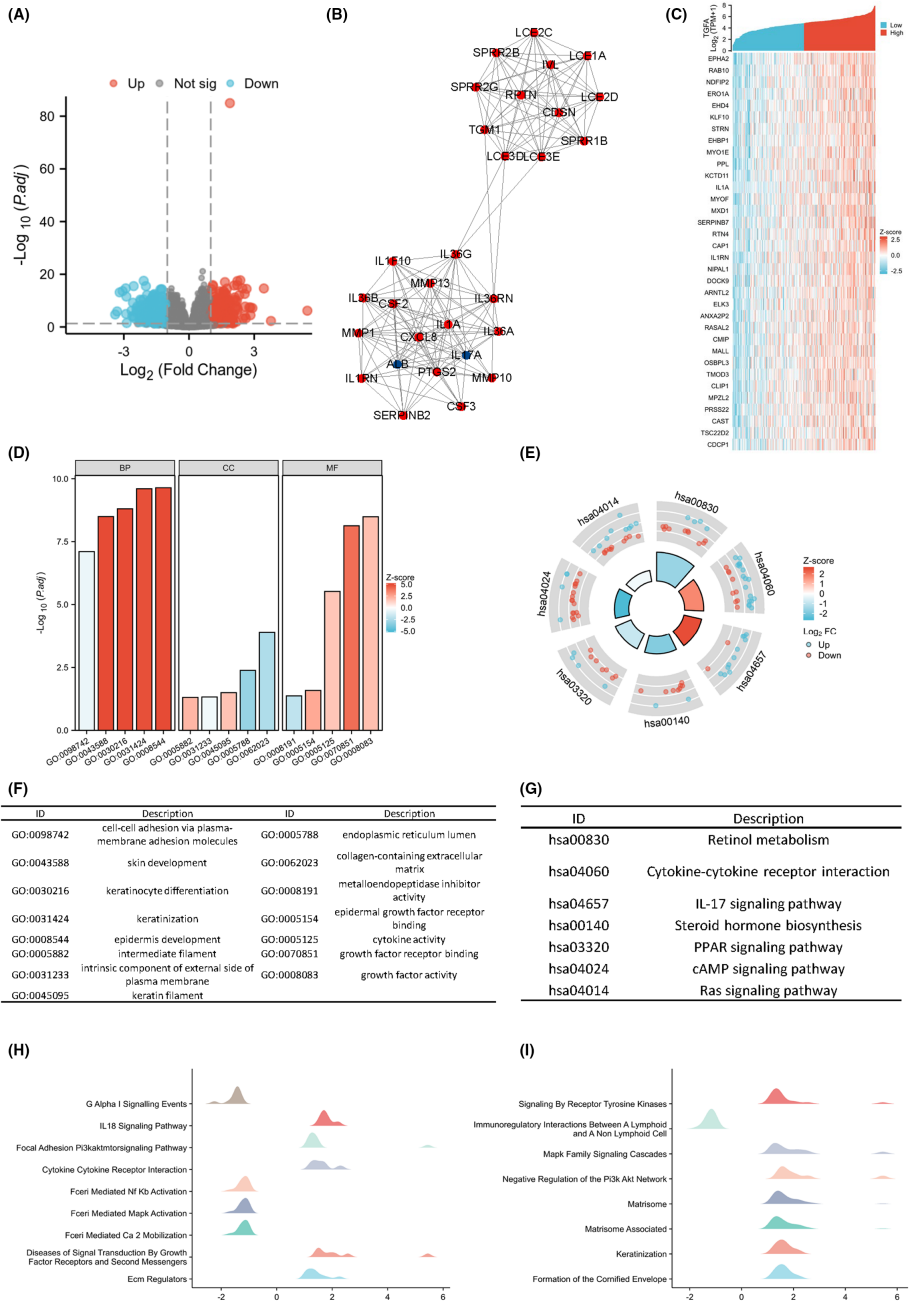
Analysis of transforming growth factor α (TGFA)‐related differentially expressed genes. (A) Volcano map by TGFA single gene difference analysis. (B) Protein interaction network diagram of HUB gene. (C) Heat maps of co‐expression of the top 35 genes most associated with TGFA in single gene correlation analysis. (D) Gene Ontology (GO) analysis results. (E) Kyoto Encyclopedia of Genes and Genomes (KEGG) analysis results. (F, G) GO and KEGG analysis class names correspond to GO and KEGG identifiers. (H, I) Gene set enrichment analysis (GSEA) results. When the horizontal coordinate is positive, TGFA expression is positively correlated with this pathway, and when the horizontal coordinate is negative, transforming growth factor α (TGFA) expression is opposite to this pathway.

The analysis results of single gene differences are shown in Figure 3A volcano map. There were 776 differentially expressed genes, including 293 highly expressed genes and 483 low‐expressed genes. These 776 genes were imported into the STRING database to construct the differential protein interaction network, the protein–protein interaction network (PPI), as shown in Figure S3. A total of 29 HUB genes (SPRR2B, CDSN, SPRR2G, RPTN, LCE2D, LCE2C, IVL, SPRR1B, TGM1, LCE3D, LCE1A, LCE3E, IL36RN, IL36G, MMP13, ALB, IL1F10, IL36A, M) were identified (MP10, PTGS2, IL17A, CXCL8, IL36B, MMP1, IL1A, CSF2, CSF3, SERPINB2, IL1RN), as shown in Figure 3B. After that, we used the correlation analysis of the 35 genes most strongly associated with TGFA to draw the co‐expression heat map, as shown in Figure 3C.

KEGG pathways and GO enrichment analyses are used to assess their potential involvement in specific molecular and biological processes. We used TGFA and its differentially expressed genes for functional enrichment analysis of GO and KEGG. GO functional enrichment analysis showed that in terms of ‘biological processes’, such pathways as cell–cell adhesion through plasma membrane adhesion molecules, skin development, keratinocyte differentiation, keratinization and epidermal development were involved. In terms of ‘cellular components’, there are abundant pathways such as intermediate filaments, intrinsic components outside the plasma membrane, keratin filaments, endoplasmic tubes and extracellular matrix (ECM) containing collagen. In terms of ‘molecular function’, the pathways such as metal endopeptidase inhibitor activity, epidermal growth factor receptor binding, cytokine activity, growth factor receptor binding and growth factor activity were significantly enriched, and the results are shown in Figure 3D,F and Table S3. The KEGG functional enrichment analysis results showed that retinol metabolism, cytokine‐cytokine receptor interaction, the IL‐17 signalling pathway, steroid hormone biosynthesis, PPAR signalling pathway, cAMP signalling pathway, Ras signalling pathway and other functional enrichment are involved, as shown in Figures 3E,G and Table S3.

Finally, GSEA functional enrichment analysis was used to predict the function of TGFA in CESC development. We found that TGFA interacts with Reactome G Alpha I Signalling Events. Reactome Fceri Mediated NF‐kB Activation, Reactome Fceri Mediated MAPK Activation, Reactome Fceri Mediated Ca2 Mobilization, Reactome Diseases of Signal Transduction by Growth Factor Receptors and Second Messengers, naba ECM regulators and other pathways are depicted in Figure 3H,I.

### 3.4 TGFA levels are associated with tumour immune cell infiltration

**FIGURE 4:**
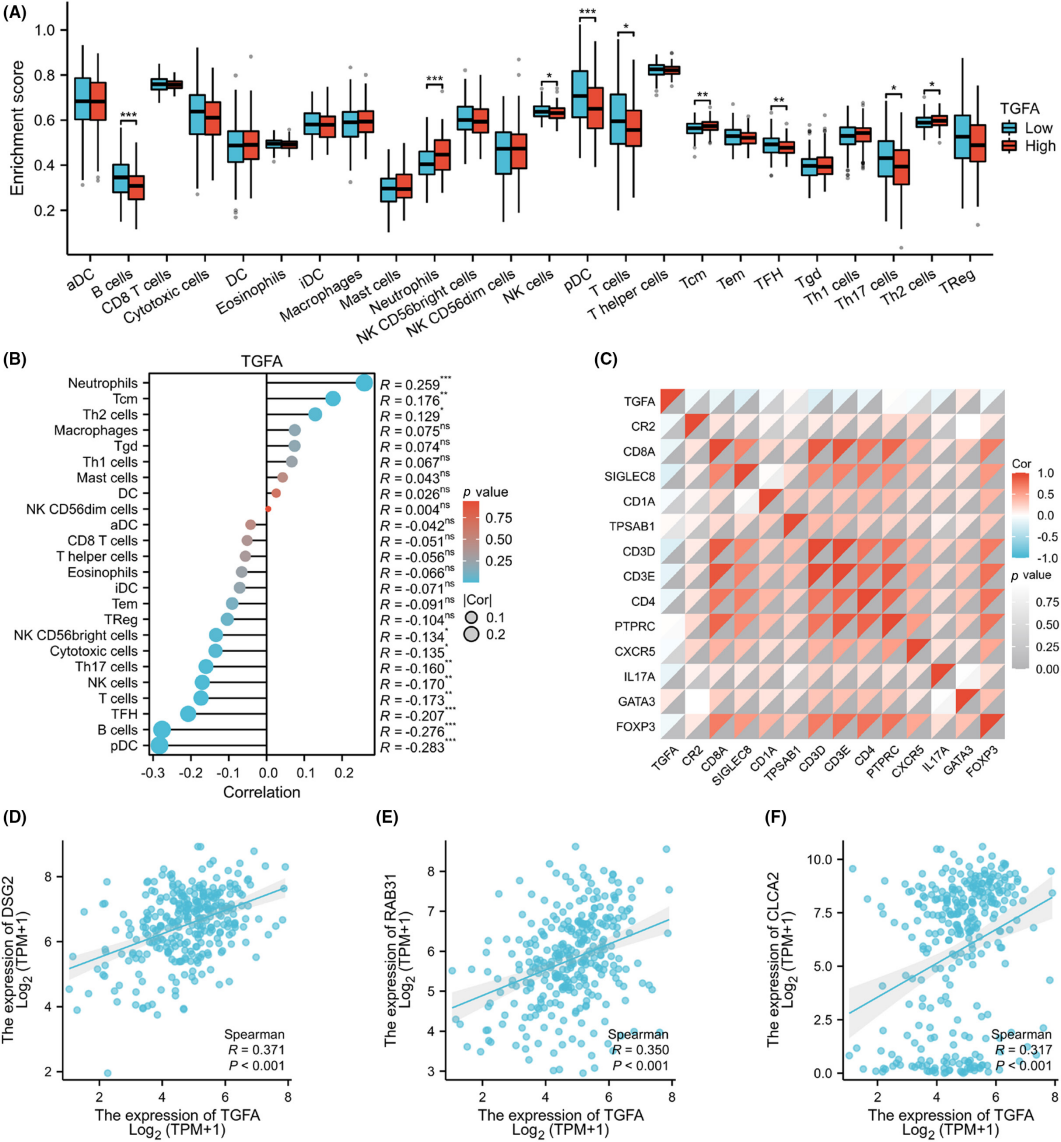
Transforming growth factor α (TGFA) levels are associated with tumour immune cell infiltration. (A) Infiltration levels of 24 types of immune cells in samples with different TGFA expression levels. (B) Analysis of the correlation between TGFA and 24 kinds of immune cell infiltration levels. (C) Thermogram of correlation between TGFA expression and surface labelled proteins of various immune cells:CR2 (B cells), CD8A(cytotoxic cells), SIGLEC8(eosinophils), CD1A (iDCs), TPSAB1(mast cells), B3GAT1 (NK cells), and IL3RA (pDCs), CD3G (T cells), CD3D (T cells), CD3E (T cells), CD4 (T helper cells), PTPRC (Tcm), CXCR5 (Tfh), IL17A (Th17), GATA3 (Th2) and FOXP3 (Treg). (D–F) Correlation analysis of TGFA with DSG2, RAB31 and CLCA2 expression levels. Significance identifier: ns (no significance), *p* ≥ 0.05; *p < 0.05; **p < 0.01; ***p < 0.001.

The relationship between TGFA expression and the degree of infiltration of 24 kinds of immune cells was analysed, and the results are shown in Figure 4A,B. The results showed that TGFA expression was positively correlated with the degree of infiltration of neutrophils and Tcm cell, and negatively correlated with the degree of infiltration of pDCs, B cells, TFH and NK cells. To verify the results of ssGSEA, we analysed the correlation between TGFA expression and the expression of various surface‐labelled proteins of immune cells and drew a heat map, as shown in Figure 4C. Heat maps showed that TGFA was strongly correlated with CR2, CD8A, SIGLEC8, CD3D, CD3E, CD4, IL17A, GATA3 and FOXP3, which was consistent with previous analyses. Finally, as shown in Figure 4D–F, we analysed the relationship between some common immunotherapy targets and TGFA and found that the expression of TGFA was significantly correlated with the expression of DSG2, Rab31 and CLCA2.

### 3.5 Functional validation of TGFA in CESC cell lines

**FIGURE 5:**
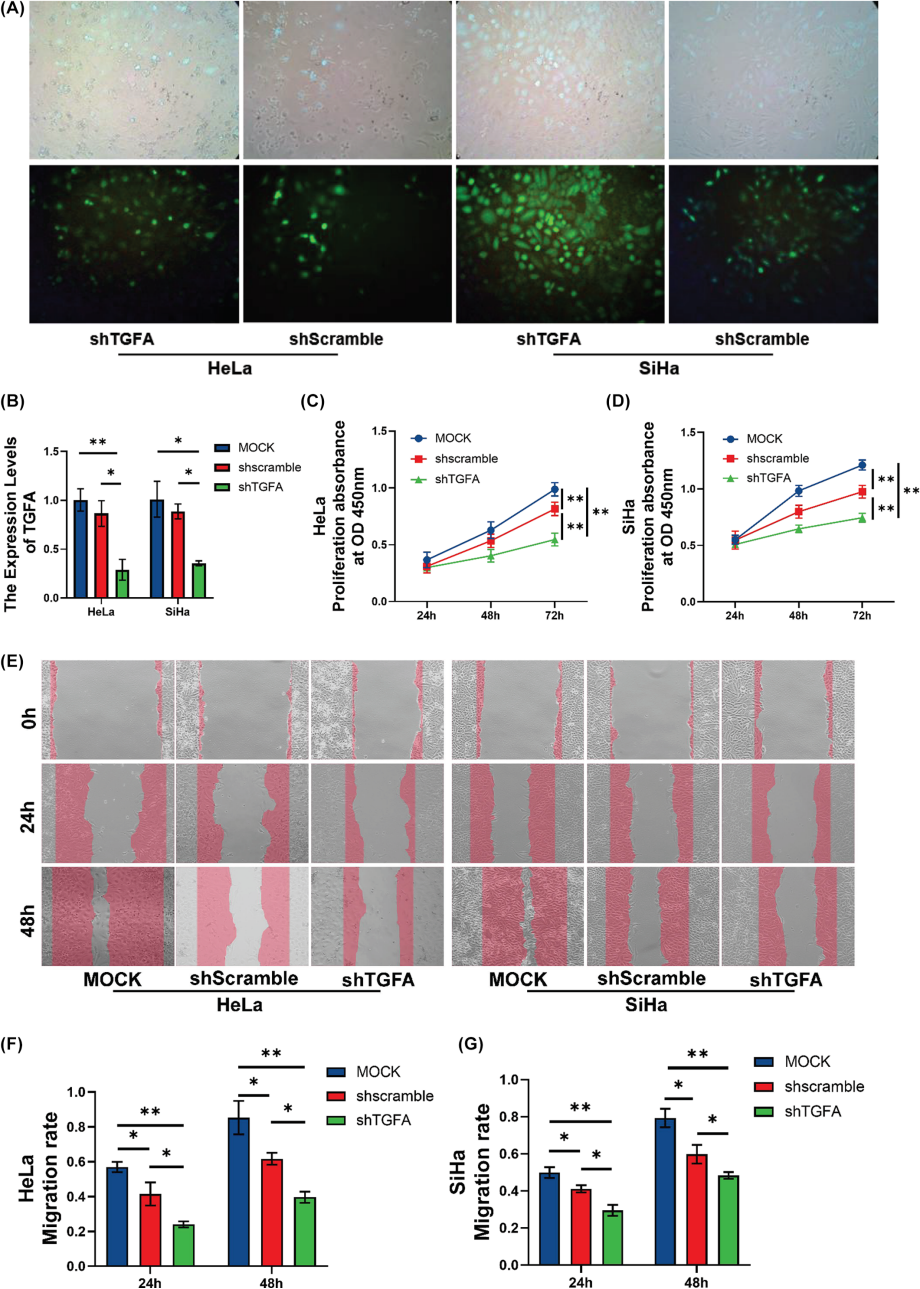
Functional validation of TGFA in CESC cell lines. (A) Fluorescence maps of lentivirus transfected HeLa and SiHa cells. (B) HeLa and SiHa cells were transfected with shTGFA, and the expression level of TGFA was detected by qRT‐PCR. (C, D) The proliferation of HeLa and SiHa cells was detected by CCK‐8. (E–G) The ability of HeLa and SiHa cells to metastasize was examined by wound healing assay. Significance identifier: ns (no significance), *p* ≥ 0.05; *p < 0.05; **p < 0.01; ***p < 0.001.

**FIGURE 6:**
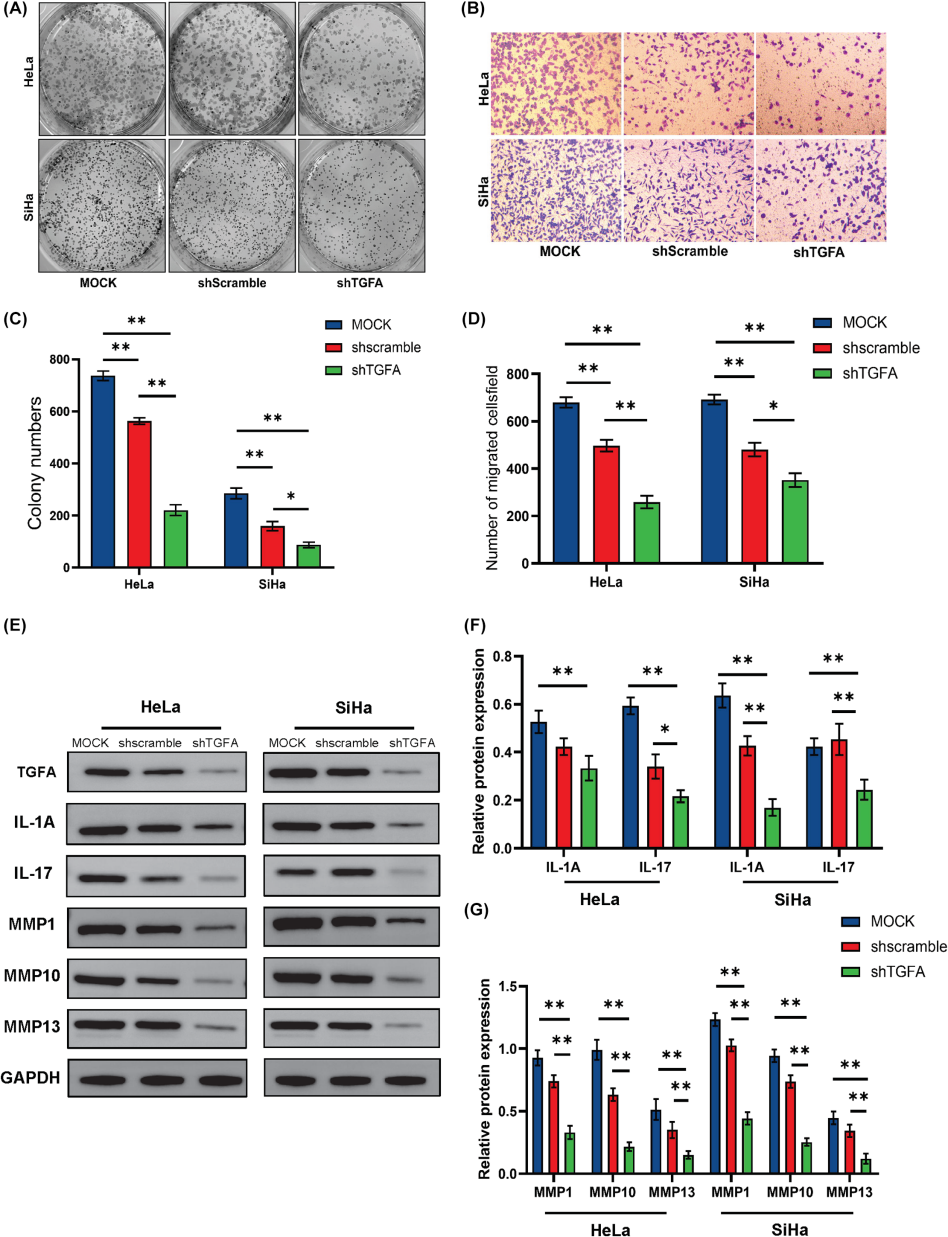
Functional validation of TGFA in CESC cell lines. (A, C) The proliferation of HeLa and SiHa cells was detected by colony formation assay. (B, D) The invasion ability of HeLa and SiHa cells was detected by Transwell assay. (E–G) The expression of IL‐1A, IL‐17, MMP1, MMP10 and MMP13 after TGFA expression reduction was detected by western blot. Significance identifier: ns (no significance), *p* ≥ 0.05; *, *p* < 0.05; **p < 0.01; ***p < 0.001.

HeLa and SiHa cells were transfected with lentivirus and screened with purinomycin for 5 days. The transfection efficiency was over 90% under a fluorescence microscope, as shown in Figure 5A. After knocking down TGFA expression in HeLa and SiHa cell lines, we confirmed that TGFA expression was significantly reduced by qPCR and western blot, as shown in Figures 5B, 6E. Then we conducted the CCK‐8 experiment and found that cell proliferation was significantly reduced after TGFA knockdown, as shown in Figure 5C,D. We also conducted wound healing experiments and found that over time, the ability of cells to transfer after TGFA knockdown was significantly weaker than that of the control group, as shown in Figure 5E‐G.

Then we conducted colony formation experiments and found that the colony formation ability decreased after the TGFA expression decreased, as shown in Figure 6A,C. The results of the Transwell experiment showed that the invasion ability of cells was also significantly weakened after TGFA knockdown, as shown in Figure 6B,D. Finally, since the expression of TGFA is closely related to IL‐17 and the MMP family, we conducted western blot experiments and found that the decreased expression of TGFA will indeed lead to the down‐regulation of IL‐1A and IL‐17. Meanwhile, the decreased expression of TGFA will also lead to the decreased expression of MMP1, MMP10 and MMP13, as shown in Figure 6E‐G.

## 4 DISCUSSION

Despite substantial advances in studies employing HPV vaccinations to prevent cervical cancer in recent years, cervical cancer is still a serious public health issue. Many people suffer from tumour invasion and metastasis, which leads to a bad prognosis and even death. As a result, it is critical to do research on the molecular processes that drive cervical cancer invasion and metastasis. More researchers are uncovering novel cancer progression indicators by examining microarray datasets in public databases and extracting valid information as bioinformatics and high‐throughput technology evolve, emphasising the importance of evaluating cervical cancer gene expression profiles to better evaluate clinical outcomes.

TGFA expression was shown to be up‐regulated in CESC in this work, which used RNA‐seq data from the TCGA and GEO databases. This shows that TGFA is linked to the onset and progression of CESC. Further analysis by IHC of 16 normal cervix, 6 cases of cervical precancerous lesions (CIN‐III) and 42 cases of cervical cancer showed that the expression of TGFA protein in tumour tissues was higher than that in normal cells, while there was no significant increase in precancerous lesions, indicating that TGFA may play a role in cervical cancer transformation. This result is consistent with the results of previous studies.^33^ Furthermore, the AUC of the ROC diagnostic curve was 0.967, showing that TGFA can be employed in the diagnosis of CESC. And the level of expression was connected to age and menopausal status. These findings imply that TGFA might be a valuable molecular marker for CESC diagnosis and prognosis in CESC patients.

To further understand the significance of TGFA in the beginning and development of CESC, single gene difference and single gene correlation analyses were used to identify 29 HUB genes closely linked to TGFA function, as well as the 35 most important genes. It consists of the SPRR protein family, the IL protein family, the MMP protein family, the CSF protein family, the TGM protein family, the IVL protein family and the RPTN protein family. To better understand the molecular mechanism of TGFA in tumour growth, TGFA and the genes related to it were shown to be enriched in GO, KEGG and GSEA. TGFA was shown to contribute to cell–cell adhesion via biological processes such as plasma membrane adhesion, keratinocyte development and keratin filaments, according to GO analysis. According to KEGG, TGFA function is linked to the IL‐17 signalling pathway, steroid hormone production, the RAS signalling system and other pathways. The GSEA results also show that TGFA is significantly enriched in many pathways associated with keratosis. In addition, TGFA is strongly associated with the functions of MAPK, NF‐kB, ECM regulators and PI3K‐AKT pathways.

A small proline‐rich protein (SPRR) that encodes a conserved set of keratinized envelope (CE) proteins that are part of the human epidermal differentiation complex (EDC) and encodes the precursor proteins of the keratinized cell envelope.^34^ SPRR has been linked to enhanced epithelial proliferation and malignant processes in several studies, implicating it in carcinogenesis and cancer formation. For example, SPRR2 is altered differently in different squamous cell carcinomas due to carcinogenic transformation.^35^ Previous studies have found that SPRR2B is highly expressed in gastric cancer and is an independent predictor of the poor prognosis of gastric cancer.^36^ The GO study revealed that TGFA is also engaged in biological processes such as keratinocyte differentiation and keratin filaments. Keratin is a cytoskeletal protein found in epithelial cells that has a role in tumour cell death, proliferation and migration. The elevated level of keratin in the serum or tumour tissue of tumour patients has been used for clinical diagnosis of tumours, and its expression level is negatively correlated with the survival of tumour patients and can be used as a prognostic indicator.^37^, ^38^ In accordance with the conclusions of the GO analysis, the GSEA data revealed a high enrichment of TGFA in numerous keratinization‐related pathways. TGFA is linked to the HUB gene, and GO and KEGG analyses revealed that TGFA is linked to keratin and keratinocyte differentiation. As a result, TGFA may be involved in controlling tumour cell proliferation and migration via this mechanism.

Previous studies have provided substantial evidence of the role of inflammation and inflammation‐related pathways in the pathogenesis of many human cancers, including cervical cancer.^39^, ^40^, ^41^ According to research, the inflammatory microenvironment is made up of tumour cells, stromal cells, immune cells, and inflammatory cells. All of these components interact closely to produce chemokines, growth factors and adhesion molecules, which further contribute to the onset and progression of many cancers.^42^, ^43^ The TGFA‐related HUB gene and KEGG functional enrichment revealed that TGFA is connected to the IL‐17 signalling pathway and the IL protein family. IL‐1A is a key inflammatory signalling cytokine secreted by various types of cells, such as monocytes, macrophages, neutrophils, smooth muscle cells, fibroblasts and cervical epithelium.^44^ IL‐1A is crucial for the development, invasion and metastasis of cancer. Previous studies have found that IL‐1A has increased expression in cervical cancer,^45^ breast cancer,^46^ pancreatic cancer,^47^ head and neck cancer^48^ and other cancers. Furthermore, elevated IL‐1A expression is linked to a bad prognosis for cancer. At the same time, in Chinese women, IL17 gene polymorphism is associated with susceptibility, positive embolization of peritumor intravascular carcinoma, and a high clinical stage of cervical cancer.^49^ IL‐17 may have an immune‐enhancing function in high‐risk HPV infection, especially in the cervical microenvironment, contributing to the disease development of its associated cervical lesions.^50^ At the same time, studies have shown that cancer‐related inflammation promotes tumour growth by assisting cancer cell survival, increasing angiogenesis, and decreasing anti‐tumour immunity.^51^ In the study of the tumour immune microenvironment of leukaemia patients, scientists found that tumour‐associated leukocytosis also showed higher levels of CXCR2 chemokines, CSF2 and CSF3.^52^ At the same time, we verified by western blotting that when TGFA expression decreased, the expression of IL‐1A and IL‐17 also decreased. These findings imply that TGFA may play a role in tumour genesis and progression by acting in inflammation‐related pathways and suppressing anti‐tumour immunity.

Furthermore, TGFA is connected to the MMP protein family. Matrix proteinases, or MMP (Matrix metalloproteinases), are metalloproteinases extracted from the matrix and are part of the subgroup of zinc endogenous proteinases produced by soft tissues. The ECM, whose degradation is a critical stage in tumour invasion and metastasis, is destroyed by the activation of matrix metalloproteinases (MMPs), which is then followed by other physiological processes including angiogenesis, apoptosis and the development of new soft tissues. All of these contribute to the spread of cancer.^53^ Squamous cell carcinoma (SCC) development and occurrence are also linked to MMP. MMP genes related to SSC include MMP1, 2, 3, 7, 9, 10, 11, 12, 13 and MMP genes are overexpressed in SCC tissues compared with normal tissues.^54^ Coincidentally, the HUB genes associated with TGFA in this study included MMP1, 10 and 13. At the same time, we verified by western blot that when TGFA expression was reduced, the expressions of MMP1, MMP10 and MMP13 were also significantly decreased. These findings imply that TGFA may contribute to tumour invasion and metastasis via proteins linked to MMP.

The Ras signalling pathway, MAPK, NF‐kb, PI3K‐AKT and other signalling pathways were all linked to TGFA, according to KEGG and GSEA analyses. Studies have shown that oestrogen is a steroid hormone, and 16α‐hydroxylation of oestrogen can increase the transcription of high‐risk papillomavirus.^50^ The Ras pathway is linked to the growth of cervical cancer. Previous studies have shown that PDL1 promotes cervical cancer development by activating RAS pathway, while DUSP7 prevents cervical cancer progression by inactivating RAS pathway.^55^, ^56^ Previous studies have found that stimulation of MAPK signal transduction pathway can promote the proliferation and metastasis of cervical cancer cells.^57^ The nuclear factor b (NF‐kB) family of transcription factors controls inflammation and immune responses in intricate and important ways. NF‐kB activation can stimulate the production of AID and APOBEC proteins, thereby establishing a molecular link between the NF‐kB pathway and the mutagenicity of cervical cancer.^58^ The PI3K/Akt/mTOR signalling system regulates several cellular and molecular events involved in tumour genesis, invasion and metastasis. Activation of the PI3K/Akt signalling pathway has been associated with a variety of human malignancies, including breast cancer, ovarian cancer, endometrial cancer and glioblastoma.^59^ These findings imply that TGFA is linked to the onset and progression of cervical cancer.

These findings imply that TGFA upregulation may promote the development of cervical cancer. In addition, TGFA was significantly correlated with the new targets of DSG2, Rab31 and CLCA2. DSG2 expression is associated with a poor prognosis and promotes the occurrence of early cervical cancer.^60^ Rab31 promotes invasion and metastasis of cervical cancer cells by inhibiting the degradation of MAPK6.^61^ CLCA2 inhibits the proliferation, migration and invasion of cervical cancer.^62^ These findings suggest that TGFA might be a therapeutic target for tumour therapy, although further proteomics and larger sample size studies are needed to prove this potential in the future.

Finally, in order to verify the function of TGFA, the expression of TGFA was down‐regulated by stable transfection of shRNA using the cervical cancer cell lines HeLa and SiHa. Down‐regulated expression of TGFA can decrease the viability of tumour cells and decrease the ability of metastasis and invasion of tumour cells. These findings support the notion that TGFA is linked to tumour incidence and development, as well as tumour cell invasion and metastasis. As a result, TGFA has the potential to be exploited as a molecular target for tumour treatment.

In conclusion, reducing TGFA expression can reduce the incidence, metastasis and invasiveness of CESC while enhancing the anti‐tumour immune response. As a consequence, TGFA can be employed as an independent risk factor for CESC as well as a feasible molecular marker for CESC diagnosis, therapy, and prognosis prediction, allowing doctors to build a more personalized treatment plan for their patients.

At the same time, this study also has many limitations. First, there is a difference between the number of cervical cancer samples and the normal number of samples, and more research is needed to narrow the difference in the number of samples. In addition, a single biomarker is unlikely to be accurate enough for prediction and diagnosis. Therefore, in the future, a combination of several different biomarkers will need to be further investigated to identify algorithms with higher diagnostic capabilities. Second, although this study established the activity of TGFA on CESC cells, further in vitro and in vivo validation of the pathway enrichment data is required. Finally, because this study was conducted retrospectively, more prospective studies are needed to eliminate the inherent bias of retrospective surveys.

## AUTHOR CONTRIBUTIONS

**Xiaoxuan Ma:** Data curation (equal); writing – original draft (equal); writing – review and editing (equal). **Jingying Zheng:** Resources (equal); writing – original draft (equal). **Kang He:** Methodology (equal); supervision (equal); writing – review and editing (equal). **Liangjia Wang:** Writing – review and editing (equal). **Zeyu Wang:** Writing – original draft (equal). **Kai Wang:** Writing – review and editing (equal). **Zunlong Liu:** Writing – review and editing (equal). **Zhiqiang San:** Writing – review and editing (equal). **Lijing Zhao:** Conceptualization (equal); supervision (equal). **Lisheng Wang:** Conceptualization (equal); supervision (equal).

## FUNDING INFORMATION

This study was supported by a grant from the Jilin Provincial Department of Science and Technology project (grant number: 20210204200YY).

## CONFLICT OF INTEREST STATEMENT

The authors declare that they have no competing interests.

## Supporting information


Data S1.

## Data Availability

Publicly available datasets were analysed in this study. This data can be found here: The TCGA database (https://portal.gdc.cancer.gov/), GSE6791 and GSE7410 of The GEO database (https://www.ncbi.nlm.nih.gov/geo/).
