# Supplementary material for: TGFA expression is associated with poor prognosis and promotes the development of cervical cancer
Source: J Cell Mol Med. 2023 Dec 28;28(3):e18086. doi: 10.1111/jcmm.18086 (PMC10844698; doi:10.1111/jcmm.18086)
Supplement: Supplementary file 1 — Data S1. [file JCMM-28-e18086-s001.docx]

| Category | No. Paired samples | | No. Non-paired samples | |
| --- | --- | --- | --- | --- |
|  | **Adjacent** | **Tumor** | **Normal** | **Tumor** |
| ACC |  |  |  | 79 |
| BLCA | 19 | 19 | 19 | 412 |
| BRCA | 113 | 113 | 113 | 1113 |
| CESC | 3 | 3 | 3 | 306 |
| CHOL | 8 | 8 | 9 | 35 |
| COAD | 41 | 41 | 41 | 480 |
| DLBC |  |  |  |  |
| ESCA | 8 | 8 | 11 | 163 |
| GBM |  |  | 5 | 169 |
| HNSC | 43 | 43 | 44 | 504 |
| KICH | 24 | 24 | 25 | 65 |
| KIRC | 72 | 72 | 72 | 541 |
| KIRP | 32 | 32 | 32 | 291 |
| LAML |  |  |  | 150 |
| LGG |  |  |  | 532 |
| LIHC | 50 | 50 | 50 | 375 |
| LUAD | 58 | 58 | 59 | 539 |
| LUSC | 49 | 49 | 49 | 502 |
| MESO |  |  |  | 87 |
| OV |  |  |  | 381 |
| PAAD | 4 | 4 | 4 | 179 |
| PCPG | 3 | 3 | 3 | 184 |
| PRAD | 52 | 52 | 52 | 501 |
| READ | 9 | 9 | 10 | 167 |
| SARC | 2 | 2 | 2 | 263 |
| SKCM | 1 | 1 | 1 | 472 |
| STAD | 27 | 27 | 32 | 375 |
| TGCT |  |  |  | 156 |
| THCA | 59 | 59 | 59 | 512 |
| THYM | 2 | 2 | 2 | 120 |
| UCEC | 23 | 23 | 35 | 554 |
| UCS |  |  |  | 57 |
| UVM |  |  |  | 80 |

**Supplementary Table 1. Sample size included in the pan-cancer analysis of paired and unpaired samples**

**Supplementary Table 2. Baseline datasheet**

| characteristics | Low expression of TGFA | High expression of TGFA | pvalue |
| --- | --- | --- | --- |
| n | 153 | 153 |  |
| Pathologic N stage, n (%) |  |  | 0.691981903 |
| N0 | 64 (32.8%) | 70 (35.9%) |  |
| N1 | 31 (15.9%) | 30 (15.4%) |  |
| Age, n (%) |  |  | 0.018827253 |
| <= 50 | 84 (27.5%) | 104 (34%) |  |
| > 50 | 69 (22.5%) | 49 (16%) |  |
| Pathologic T stage, n (%) |  |  | 0.790972877 |
| T1 | 69 (28.4%) | 71 (29.2%) |  |
| T2 | 38 (15.6%) | 34 (14%) |  |
| T3 | 9 (3.7%) | 12 (4.9%) |  |
| T4 | 4 (1.6%) | 6 (2.5%) |  |
| Pathologic M stage, n (%) |  |  | 0.20357193 |
| M0 | 59 (23%) | 57 (22.3%) |  |
| M1 | 8 (3.1%) | 3 (1.2%) |  |
| MX | 59 (23%) | 70 (27.3%) |  |
| Clinical stage, n (%) |  |  | 0.587034548 |
| Stage I | 78 (26.1%) | 84 (28.1%) |  |
| Stage II | 34 (11.4%) | 35 (11.7%) |  |
| Stage III | 22 (7.4%) | 24 (8%) |  |
| Stage IV | 14 (4.7%) | 8 (2.7%) |  |
| Primary therapy outcome, n (%) |  |  | 0.123768704 |
| PD | 7 (3.2%) | 16 (7.3%) |  |
| SD | 3 (1.4%) | 3 (1.4%) |  |
| PR | 2 (0.9%) | 6 (2.7%) |  |
| CR | 95 (43.4%) | 87 (39.7%) |  |
| Weight, n (%) |  |  | 0.951567178 |
| <= 70 | 68 (24.5%) | 70 (25.3%) |  |
| > 70 | 69 (24.9%) | 70 (25.3%) |  |
| Height, n (%) |  |  | 0.064908851 |
| <= 160 | 76 (29%) | 58 (22.1%) |  |
| > 160 | 58 (22.1%) | 70 (26.7%) |  |
| Race, n (%) |  |  | 0.353659535 |
| Asian | 12 (4.6%) | 8 (3.1%) |  |
| Black or African American | 18 (6.9%) | 13 (5%) |  |
| White | 100 (38.3%) | 110 (42.1%) |  |
| BMI, n (%) |  |  | 0.768564917 |
| <= 25 | 50 (19.2%) | 50 (19.2%) |  |
| > 25 | 83 (31.9%) | 77 (29.6%) |  |
| Histological type, n (%) |  |  | 0.172153315 |
| Adenocarcinoma | 26 (8.5%) | 21 (6.9%) |  |
| Adenosquamous | 5 (1.6%) | 1 (0.3%) |  |
| Squamous cell carcinoma | 122 (39.9%) | 131 (42.8%) |  |
| Histologic grade, n (%) |  |  | 0.185271463 |
| G1 | 8 (2.9%) | 11 (4%) |  |
| G2 | 63 (23%) | 72 (26.3%) |  |
| G3 | 68 (24.8%) | 51 (18.6%) |  |
| G4 | 1 (0.4%) | 0 (0%) |  |
| Menopause status, n (%) |  |  | 0.016852106 |
| Pre | 53 (22.7%) | 73 (31.3%) |  |
| Peri | 17 (7.3%) | 8 (3.4%) |  |
| Post | 47 (20.2%) | 35 (15%) |  |

**Supplementary Table 3. GO and KEGG analysis**

| ONTOLOGY | ID | Description | GeneRatio | BgRatio | pvalue | p.adjust | qvalue |
| --- | --- | --- | --- | --- | --- | --- | --- |
| BP^a^ | GO:0008544 | epidermis development | 49/722 | 355/18800 | 6.62868E-15 | 2.95639E-11 | 2.48052E-11 |
| BP | GO:0031424 | keratinization | 23/722 | 85/18800 | 7.15426E-14 | 1.5954E-10 | 1.3386E-10 |
| BP | GO:0043588 | skin development | 42/722 | 296/18800 | 2.31168E-13 | 2.76754E-10 | 2.32207E-10 |
| cBP | GO:0030216 | keratinocyte differentiation | 31/722 | 167/18800 | 2.4821E-13 | 2.76754E-10 | 2.32207E-10 |
| BP | GO:0098742 | cell-cell adhesion via plasma-membrane adhesion molecules | 38/722 | 279/18800 | 1.15731E-11 | 7.52671E-09 | 6.31519E-09 |
| CC^a^ | GO:0062023 | collagen-containing extracellular matrix | 42/776 | 429/19594 | 6.92611E-08 | 2.50184E-05 | 2.14284E-05 |
| CC | GO:0005788 | endoplasmic reticulum lumen | 29/776 | 311/19594 | 1.86327E-05 | 0.000570063 | 0.000488262 |
| CC | GO:0045095 | keratin filament | 12/776 | 102/19594 | 0.000704553 | 0.012447096 | 0.010660993 |
| CC | GO:0005882 | intermediate filament | 19/776 | 216/19594 | 0.00100327 | 0.01575506 | 0.01349428 |
| CC | GO:0031233 | intrinsic component of external side of plasma membrane | 5/776 | 24/19594 | 0.002182646 | 0.02721888 | 0.023313092 |
| MF^a^ | GO:0008083 | growth factor activity | 30/752 | 162/18410 | 3.05839E-12 | 7.23819E-10 | 5.98801E-10 |
| MF | GO:0070851 | growth factor receptor binding | 27/752 | 139/18410 | 1.17382E-11 | 1.66683E-09 | 1.37893E-09 |
| MF | GO:0005125 | cytokine activity | 31/752 | 235/18410 | 8.68624E-09 | 6.16723E-07 | 5.10202E-07 |
| MF | GO:0005154 | epidermal growth factor receptor binding | 6/752 | 32/18410 | 0.001660985 | 0.017187373 | 0.014218761 |
| MF | GO:0008191 | metalloendopeptidase inhibitor activity | 4/752 | 16/18410 | 0.003392257 | 0.030878239 | 0.025544933 |
| KEGG^a^ | hsa00830 | Retinol metabolism | 14/329 | 68/8164 | 3.87073E-07 | 2.12116E-05 | 1.76017E-05 |
| KEGG | hsa04060 | Cytokine-cytokine receptor interaction | 27/329 | 295/8164 | 5.32271E-05 | 0.001220631 | 0.001012894 |
| KEGG | hsa04657 | IL-17 signaling pathway | 13/329 | 94/8164 | 9.19024E-05 | 0.001798662 | 0.001492551 |
| KEGG | hsa00140 | Steroid hormone biosynthesis | 10/329 | 61/8164 | 0.000142221 | 0.002292272 | 0.001902154 |
| KEGG | hsa03320 | PPAR signaling pathway | 11/329 | 75/8164 | 0.000186901 | 0.002845055 | 0.00236086 |
| KEGG | hsa04024 | cAMP signaling pathway | 20/329 | 221/8164 | 0.000578287 | 0.0083395 | 0.006920215 |
| KEGG | hsa04014 | Ras signaling pathway | 19/329 | 235/8164 | 0.002946636 | 0.031195553 | 0.025886436 |

^a^ CC, Cellular Component; BP, Biological Process; MF, Molecular Function.


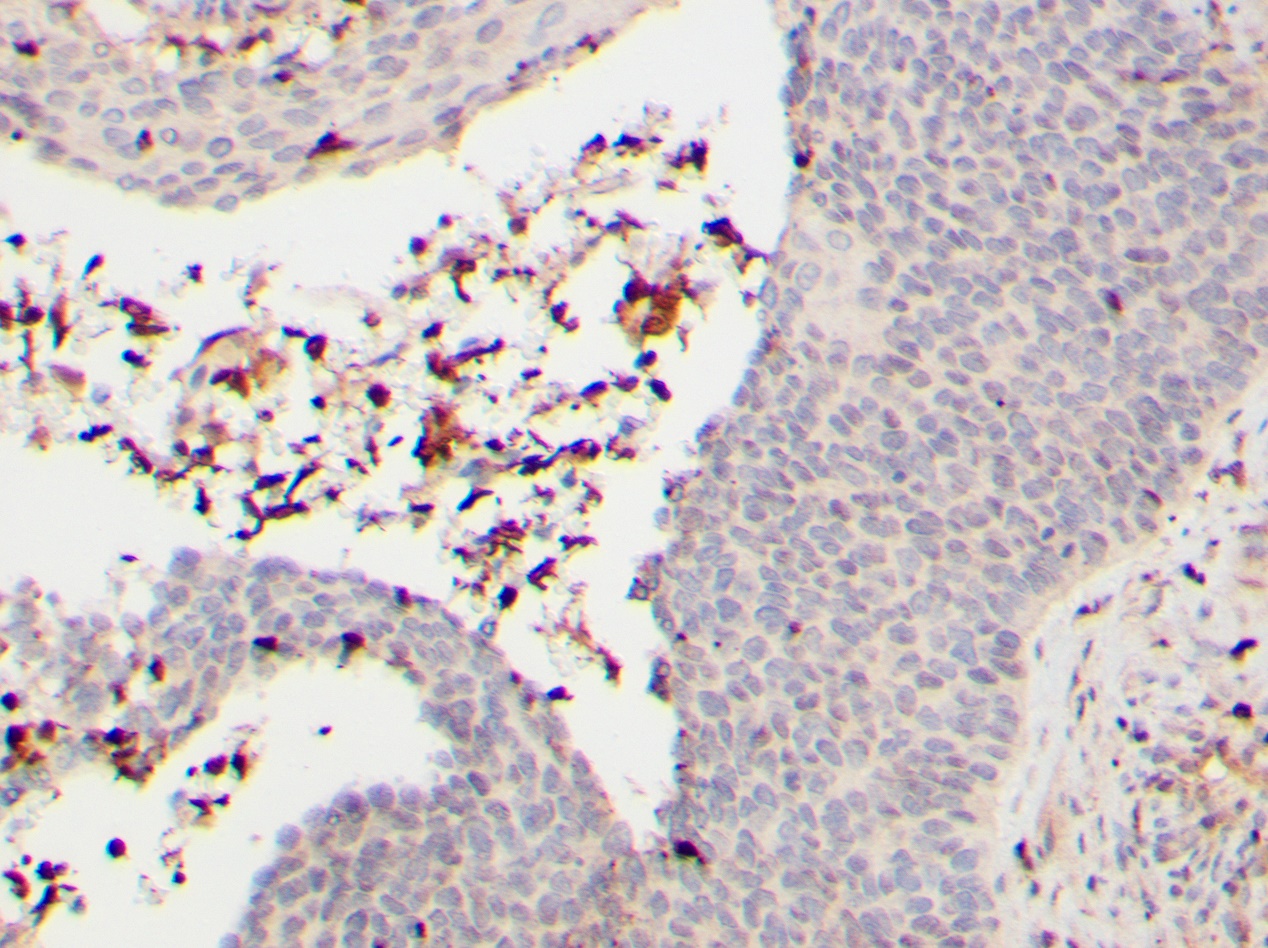
**Supplementary Figure 1. The expression of TGFA in cervical precancerous lesions (CIN-Ⅲ) was detected by immunohistochemical staining**

**Supplementary Figure 2.** **Clinical correlation analysis of TGFA in CESC.**


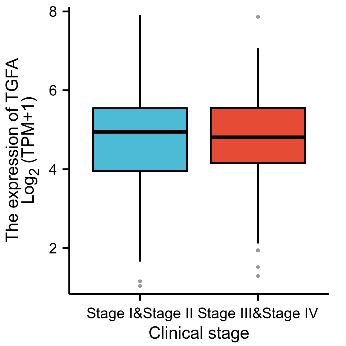

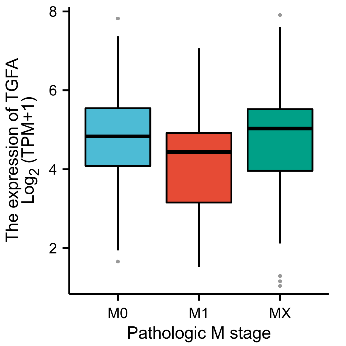

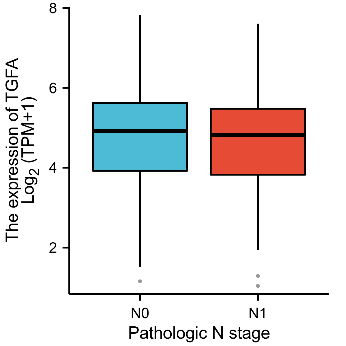

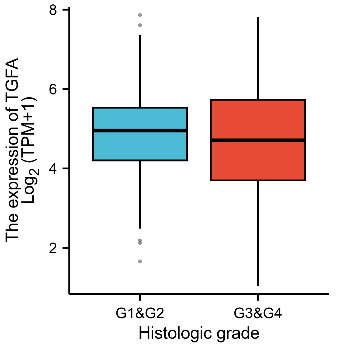

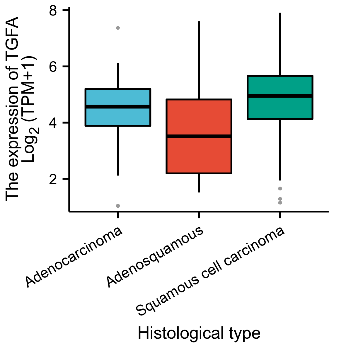

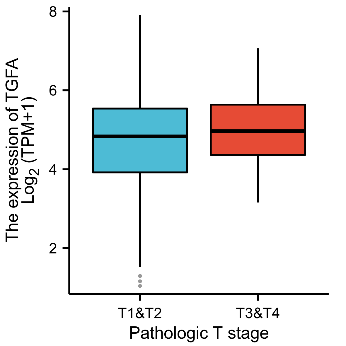


**Supplementary Figure 3.** **Protein-protein Interaction Network (PPI).**

**
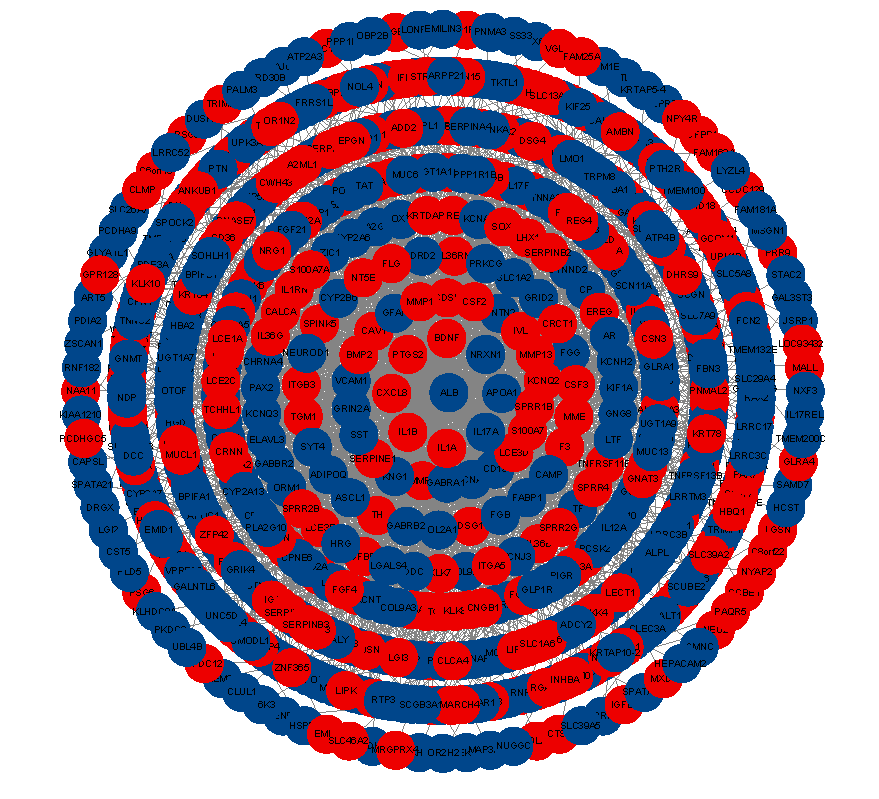
**
